# Notes and News

**Published:** 1903-01-31

**Authors:** 


### NOTES AND NEWS.

A COTTAGE HOSPITAL has been opened at Maryport, Cumberland, in memory of Queen Victoria.

THE Congress and Exhibition of the Sanitary Institute will this year be held at Bradford, commencing July 7th.

AN announcement has been made by Professors Panichi and Tizzoni to the Academy of Sciences at Bologna that they have discovered the bacillus of pneumonia, and also a serum to counteract its effects.

A MEDAL has been struck by the German Samaritans' Union in honour of Professor von Esmarck's eightieth birthday. This medal will be awarded to those who have distinguished themselves in the service of the sick.

MR. HENRY PHIPPS has given £250,000 for the establishment of a hospital for consumption at Philadelphia, with especial facilities for research in the treatment and prevention of the disease. The institution will be modelled after the organisation of the Pasteur Institute in Paris.

SIR HENRY BURDETT delivered one of his series of addresses in aid of the League of Mercy last week at Camberwell. The Mayor and Town Council lent the Camberwell Town Hall for the occasion, and it was filled by a most appreciative audience, many of whom joined the League at the conclusion of the lecture.

THE eleventh International Congress of Hygiene and Demography will be held in Brussels from the 2nd to the 8th of September, this year, under the patronage of his Majesty the King of the Belgians. The Secretary-General of the Congress is Professor Dr. Putzeys. Dr. Paul F. Moline, 42 Walton Street, Chelsea, S.W., is the Hon. Secretary of the British Committee, and will supply all information.

A COTTAGE HOSPITAL is to be erected at Nice as a memorial to Queen Victoria.

IN the German Army a permanent Dental Department has been established to attend to the requirements of the non-commissioned officers and men.

THE cholera, which has broken out at Bangkok, has already claimed two European victims in Messrs. Phillips and Holloway, masters at the King's College.

THE Harben Gold Medal for distinguished service in the cause of public health and the advancement of sanitary science has been awarded to Sir Charles Cameron, F.R.C.S., C.B.

THE Finsbury and Poplar Borough Councils join with the Kensington Borough Council in agreeing that the Orchard and Joyce Green Hospitals should be used for the treatment of consumptive patients.

DR. CLOUSTON, who has been elected President of the Royal College of Surgeons, Edinburgh, is medical superintendent of the Royal Asylum for the Insane at Morningside, and editor of the *Journal of Mental Science*.

THE presence of a considerable amount of small-pox in various parts of the country is rather disquieting. Liverpool has numbered its patients by hundreds, and has introduced the infection, in one case at any rate, into Ireland.

THE new buildings at the Wigan Corporation's Sanatorium at Whelley, for infectious diseases, have been opened and consist of wards for male and female patients suffering from scarlet fever, observation rooms, nurses' duty rooms, a discharging block for convalescent patients, administrative and domestic offices.

A VERY flagrant case of disregard of the public safety came before the magistrates at Stockport recently. Two men recovering from small-pox in the Isolation Hospital left the grounds and visited a public-house to buy drink. They were find £5, with costs. One man went to prison for a month in default of paying the fine.

THE present agitation in favour of a modified form of abstinence from alcoholic drink, confining its use exclusively to meal times, gives those who hold moderate views an opportunity to embrace the temperance cause. It cannot be supposed that such a measure will cure the habitual drunkard, but by establishing an etiquette in the matter as to when the respectable citizen may partake of a moderate amount of alcohol without provoking comment, it will render the lot of many waiverers far easier than it has been when total abstinence alone has been regarded as the path of virtue by its followers, whilst provoking in other directions a ridicule which the young are loath to face.

